# Detection of Myocardial Deformation Patterns and Prognostic Value of Routine Echocardiographic Parameters in Patients with Cardiac Sarcoidosis Versus Extracardiac Sarcoidosis: Systematic Review and Meta-Analysis

**DOI:** 10.3390/diagnostics15050518

**Published:** 2025-02-20

**Authors:** Hritvik Jain, Maryam Shahzad, Muhammad Usman, Anil KC, Jagjot Singh, Jyoti Jain, Ramez M. Odat, Aman Goyal, Faizan Ahmed, Raheel Ahmed

**Affiliations:** 1 Department of Cardiology, All India Institute of Medical Sciences, Jodhpur 342005, India; hritvikjain2001@gmail.com (H.J.); dr.jyotijain18@gmail.com (J.J.); 2 Department of Internal Medicine, Dow University of Health Sciences, Karachi 74200, Pakistan; maryamshahzad575@gmail.com (M.S.); muhammadusmantaufiq34@gmail.com (M.U.); 3 Department of Internal Medicine, Mayo Clinic in Florida, Jacksonville, FL 32224, USA; kc.anil@mayo.edu; 4 Department of Internal Medicine, Government Medical College, Amritsar 143001, India; ijagjot@gmail.com; 5 Department of Medicine, Faculty of Medicine, Jordan University of Science and Technology, Irbid 22110, Jordan; rmodat22@med.just.edu.jo; 6 Department of Internal Medicine, King Edward Memorial Hospital and Seth G.S. Medical College, Mumbai 400083, India; amanmgy@gmail.com; 7 Department of Cardiology, Duke University Medical Center, Durham, NC 27710, USA; drfaizanamalik@gmail.com; 8 Department of Cardiology, National Heart and Lung Institute, Imperial College London, London SW7 2AZ, UK

**Keywords:** sarcoidosis, cardiac sarcoidosis, speckle tracking echocardiography, Doppler echocardiography, myocardial deformation

## Abstract

**Highlights:**

**What are the main findings?**

- Patients with CS show significant reductions in LV GLS and TAPSE compared to ECS.
- 2D-STE demonstrates promising efficacy in detecting altered myocardial strain patterns in CS.

**What is the implication of the main finding?**

- These findings shall aid in the early and timely detection of CS using STE as a complementary imaging modality.
- STE should be considered in patients with suspected CS and normal LVEF.

**Abstract:**

**Background:** Sarcoidosis is a multisystem disorder characterized by non-caseating granulomas in various organs. While cardiac sarcoidosis (CS) is clinically rare, it has significant implications, including heart failure, ventricular arrhythmias, and sudden cardiac death. Speckle-tracking echocardiography has emerged as a promising tool for detecting subclinical myocardial dysfunction, which is cost-efficient and readily available. This meta-analysis aims to evaluate differences in functional echocardiographic parameters between patients with CS and extracardiac sarcoidosis (ECS) to improve early recognition and management. **Methods:** A comprehensive search of major bibliographic databases was conducted to identify studies up to December 2024. Mean differences (MDs) with 95% CIs were pooled using the inverse-variance random-effect model. **Results:** Seven studies with 478 patients with sarcoidosis (CS: 159 and ECS: 319) were included. Patients with CS had a significant reduction in left ventricular global longitudinal strain (MD: −2.73; 95% CI: −4.09, −1.38; *p* < 0.0001) and tricuspid annular plane systolic excursion (MD: −0.59; 95% CI: −1.12, −0.05; *p* = 0.03) compared to patients with ECS. No significant differences in the LV global circumferential strain, interventricular septum thickness, left ventricular ejection fraction, E/A ratio, E/E’ ratio, LV end-diastolic diameter, and LV end-systolic diameter were noted. **Conclusions:** LV GLS and TAPSE are promising parameters for the early detection of cardiac involvement in sarcoidosis, with significant prognostic implications. Although STE provides a cost-effective and accessible alternative to CMR and FDG-PET, further research is needed to standardize its use and validate diagnostic cut-offs.

## 1. Introduction

Sarcoidosis is a rare multisystem disorder presenting with a range of clinically variable symptoms. Histologically, it is characterized by non-caseating granulomas in multiple organ systems, including, but not limited to, the lungs, skin, lymphatics, and central nervous system [1,2]. Although the clinical manifestation of cardiac involvement is rare (around 5%), multiple studies assessing autopsies and histopathological findings from multiple patients have shown an asymptomatic subclinical cardiac involvement of about 25–30% [3]. The common clinical manifestations of cardiac sarcoidosis (CS) include heart failure (HF), ventricular arrhythmias, and high-grade atrioventricular (AV) node conduction diseases, depending on the area and extent of cardiac involvement [4]. Affected patients are also at an increased risk of sudden cardiac death (SCD), which may, in some cases, be the initial presentation of the disease [5]. In certain studies, cardiac involvement was associated with a significant increase in morbidity and mortality in patients, with some reporting it as the most common cause of death in patients with sarcoidosis [6,7,8]. Although the literature is extensive on concluding an increased risk of mortality among patients with cardiac involvement in sarcoidosis, prompt treatment of these patients with different immunosuppressive therapies, as well as targeted therapies for heart failure and arrhythmias, generally leads to a better prognosis, decreasing the risk of adverse cardiovascular outcomes [9,10,11]. However, a proper diagnosis of cardiac involvement is warranted before initiation of the treatment.

Classically, the gold standard for diagnosing CS remains the histopathological evidence of infiltration of the myocardium [12]. This, however, has led to underdiagnoses as the infiltration pattern is usually patchy, leading to a high number of false negatives and a sensitivity of just 20 to 36% [13]. In contrast, studies assessing imaging modalities have shown better results in diagnosing CS with a reported prevalence of around 39% [14,15]. Even though newer guidelines, like the one published by the Japanese Circulation Society [16], are shifting towards using imaging modalities for diagnosis, many guidelines still suggest a combination of these imaging methods with endomyocardial biopsies (EMBs) to increase the diagnostic yield by almost 50% [4]. The imaging modalities being considered are cardiac magnetic resonance (CMR) imaging, fluorine-18 fluorodeoxyglucose (FDG)–positron emission tomography (PET), and echocardiography. However, CMR and FDG-PET remain the fundamental imaging modalities for diagnosing CS, with multiple studies showing good sensitivity and specificity [16,17,18]. Despite its limited sensitivity and specificity, echocardiography remains widely used due to its low cost and easy accessibility.

There is an ample amount of the literature consisting of primary research assessing the changes in echocardiography parameters in patients with CS vs. patients with extracardiac sarcoidosis (ECS), which may help in diagnosing CS as well as to characterize better the extent of cardiac involvement with a notable absence of a proper meta-analysis pooling data from all these studies. Thus, in this meta-analysis, we pool data from these studies describing the changes in the echocardiographic parameters in patients with CS vs. ECS.

## 2. Methods

This meta-analysis followed the PRISMA 2020 guidelines for systematic reviews and meta-analyses and the suggested procedures of the Cochrane Collaboration (Supplementary Table S1) [19]. This study was registered under the unique identifier CRD42024621276 in the PROSPERO International Prospective Register of Systematic Reviews.

### 2.1. Data Sources and Search Strategy

A comprehensive systematic review of the major electronic databases, including MEDLINE (via PubMed), Embase, COCHRANE Central, Web of Science, and Scopus, was carried out until December 2024. This search was aimed at retrieving studies evaluating echocardiography-based cardiovascular function parameters in patients with CS compared to ECS. No restrictions on the language or publication year were imposed. To create a search string, keywords such as “cardiac sarcoidosis”, “echocardiography”, “left ventricle ejection fraction”, “Doppler echocardiography”, “speckle tracking”, and “strain imaging” were used in combination with “AND” and “OR” Boolean operators. Reference lists of the included studies were manually reviewed to identify additional studies. Database-specific search strategies are detailed in Supplementary Table S2.

### 2.2. Eligibility Criteria

This systematic review and meta-analysis evaluated individual studies using the PICOS framework (population, intervention, comparison, outcomes, and study designs). Studies were included if they (a) were randomized controlled trials, case–control or cohort studies, (b) included patients with CS in one arm, (c) included patients with ECS in one arm, (d) performed echocardiography for all participants, and (e) reported one of the outcomes of interest.

The outcomes of interest included the left ventricular global longitudinal strain (LV GLS), left ventricular global circumferential strain (LV GCS), tricuspid annular plane systolic excursion (TAPSE), left ventricular ejection fraction (LVEF), interventricular septal thickness (IVST), early-to-late diastolic filling velocity ratio (E/A ratio), early mitral inflow velocity to early diastolic tissue Doppler velocity ratio (E/E’ ratio), left ventricular end-diastolic diameter (LVEDD), and left ventricular end-systolic diameter (LVESD).

Studies were excluded if they (a) were not observational or randomized controlled trials, (b) did not compare CS patients with ECS patients, or (c) did not report outcomes of interest.

### 2.3. Study Selection

All retrieved records from the systematic literature search were imported into the EndNote Reference Manager (Version X7.5) (Clarivate Analytics, Philadelphia, PA, USA). The entire selection of records underwent the removal of duplicates. Two independent reviewers (M.S. and M.U.T.) conducted preliminary screening using titles and abstracts to include relevant studies. Potentially relevant studies were considered eligible for a comprehensive full-text evaluation for inclusion. Any disagreements were resolved through consensus or by consulting a third independent reviewer (H.J.).

### 2.4. Data Extraction and Quality Assessment

Data extraction was independently performed by two authors (H.J. and J.J.) using a pre-designed Microsoft Excel spreadsheet. Any discrepancies were addressed and resolved by a third author (M.S.). The extracted data included the first author’s name, country of study, publication year, sample size, mean age, male %, type of echocardiography device used, history of steroid use, comorbidities %, and the reported outcomes. The quality of observational studies was evaluated using the Newcastle–Ottawa Scale (NOS), an eight-item tool designed to assess the quality of non-randomized studies in meta-analyses. The NOS assigns scores ranging from 0 to 9 stars, with scores of 7 or higher indicating high quality and those below 7 signifying low quality [20].

### 2.5. Data Synthesis

The data synthesis was conducted using the Nordic Cochrane Collaboration’s Review Manager (RevMan, Version 5.4.1). Effect estimates were calculated by pooling mean differences (MDs) with 95% confidence intervals (CIs) using the inverse-variance random effect model. Statistical significance was defined as a *p*-value of less than 0.05. Heterogeneity was evaluated using the Higgins’ I^2^ test, where values of 0–25% represented low heterogeneity, 25–75% indicated moderate heterogeneity, and values above 75% reflected high heterogeneity [21]. To find significant studies influencing heterogeneity, a leave-one-out sensitivity analysis was employed. Publication bias was assessed via the visual inspection of funnel plots, and no statistical tests were used because the total number of included studies was less than ten [22].

## 3. Results

**Figure 1:**
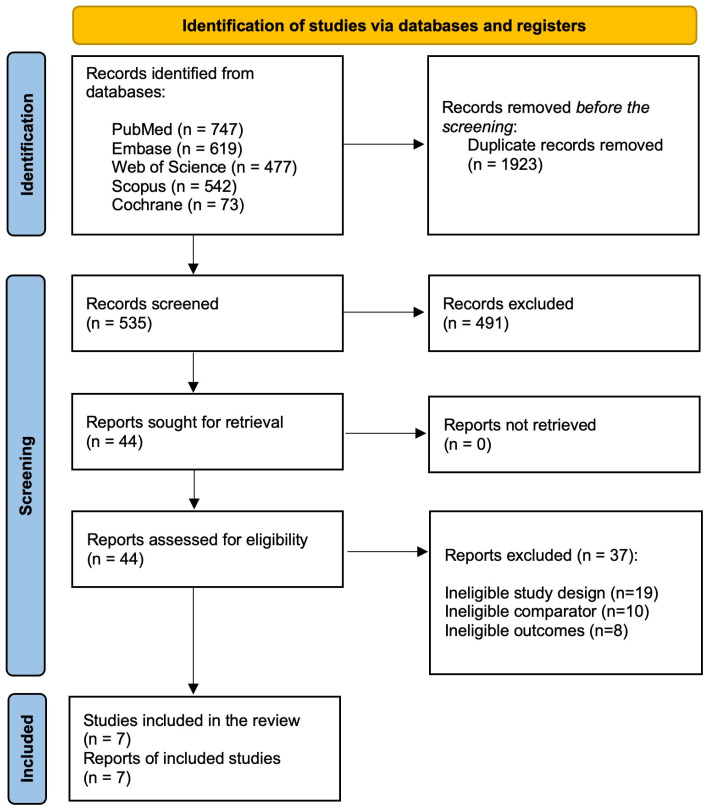
PRISMA flowchart depicting the study selection process.

The initial database search retrieved 2458 potential records. After removing duplicates (*n* = 1923), 535 records were subjected to preliminary screening. During the preliminary screening using titles and abstracts, 491 were excluded. The remaining 44 records underwent a full-text review against the predetermined inclusion criteria. During the full-text comprehensive assessment, 37 records were excluded due to the following reasons: ineligible study design (*n* = 19), ineligible comparator (*n* = 10), and ineligible outcomes (*n* = 8). Finally, seven studies were included in this meta-analysis (18–24). The entire study selection process is depicted using the PRISMA flowchart in Figure 1.

### 3.1. Study Selection and Baseline Characteristics

This systematic review and meta-analysis included a total of seven studies, encompassing 478 patients with sarcoidosis (CS: 159; ECS: 319) [23,24,25,26,27,28,29]. The mean ages of CS and ECS were 54.15 ± 10.28 and 53.58 ± 12.9 years, respectively. The studies were conducted in multiple countries—two in Japan and one each in India, Poland, the United States, Germany, and Turkey. The baseline characteristics of the included studies are reported in Table 1. The inclusion and exclusion criteria of every included study are reported in Supplementary Table S3.

### 3.2. Outcomes

#### 3.2.1. Left Ventricular Global Longitudinal Strain (LV GLS)

**Figure 2:**
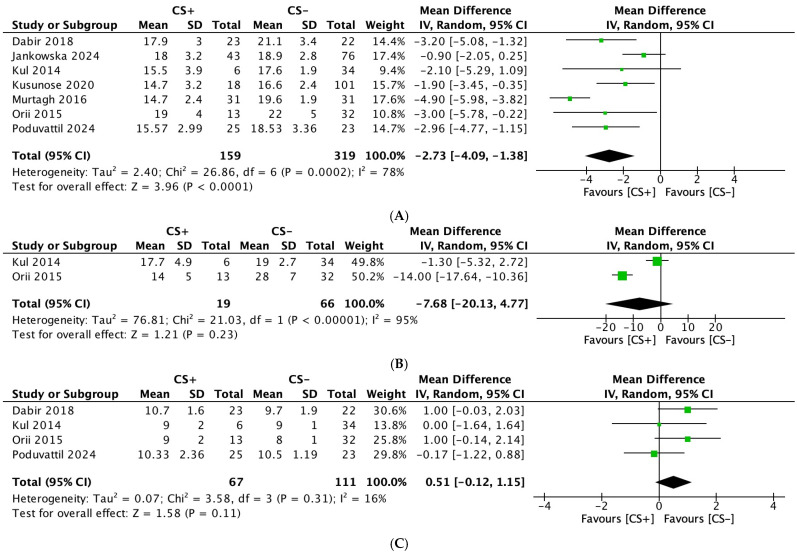
Forest plots comparing echocardiography parameters in patients with cardiac sarcoidosis (CS) to extracardiac sarcoidosis (ECS): (**A**): left ventricular global longitudinal strain; (**B**): left ventricular global circumferential strain; and (**C**): interventricular septal thickness.

The data on LV GLS were reported by all seven studies [23,24,25,26,27,28,29]. The analysis revealed a significant decrease in LV GLS in patients with CS (MD: −2.73; 95% CI: −4.09, −1.38; *p* < 0.0001) (Figure 2A). High heterogeneity was observed among the studies for this outcome (I^2^: 78%). However, after excluding Murtagh et al. [25], the I^2^ value dropped significantly to 25%.

#### 3.2.2. Left Ventricular Global Circumferential Strain (LV GCS)

Two studies [23,24], including 85 participants (CS: 19; ECS: 66), assessed LV GCS as an outcome. No significant difference was observed in LVGCS between the two groups (MD: −7.68; 95% CI: −20.13, 4.77; *p* = 0.23) (Figure 2B). High heterogeneity was noted among the studies for this outcome (I^2^: 95%).

#### 3.2.3. Interventricular Septum Thickness (mm) (IVST)

Four studies [23,24,26,28], including 178 participants (CS: 67; ECS: 111), assessed IVST as an outcome. No significant difference was observed in IVST between the two groups (MD: 0.51; 95% CI: −0.12, 1.15; *p* = 0.11) (Figure 2C). Low heterogeneity was observed among the studies for this outcome (I^2^: 16%).

#### 3.2.4. Tricuspid Annular Plane Systolic Excursion (TAPSE)

**Figure 3:**
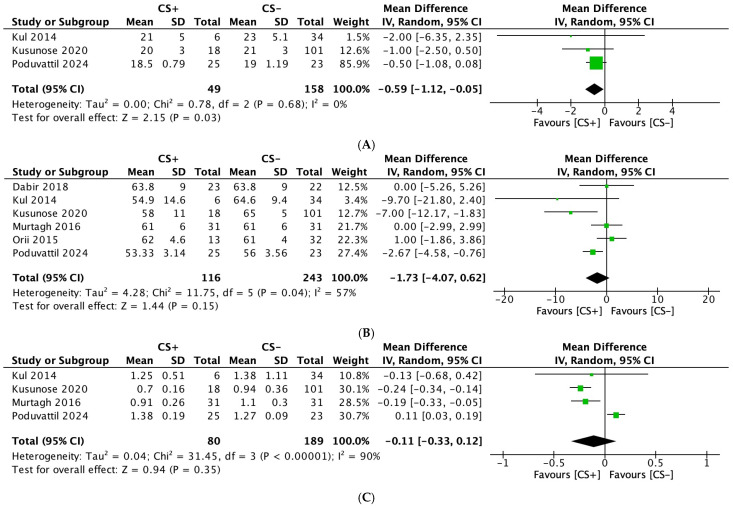
Forest plots comparing echocardiography parameters in patients with cardiac sarcoidosis (CS) to extracardiac sarcoidosis (ECS): (**A**): tricuspid annular plane systolic excursion; (**B**): left ventricular ejection fraction; and (**C**): E/A ratio.

Three studies [23,27,28], including 207 participants (CS: 49; ECS: 158), evaluated TAPSE as an outcome. The analysis showed a significant reduction in TAPSE in patients with CS (MD: −0.59; 95% CI: −1.12, −0.05; *p* = 0.03) (Figure 3A). No heterogeneity was observed among the studies for this outcome (I^2^ = 0%).

#### 3.2.5. Left Ventricular Ejection Fraction (LVEF)

Six studies [23,24,25,26,27,28], including 359 participants (CS: 116; ECS: 243), assessed the LVEF as an outcome. No significant differences in the LVEF were observed between the two groups (MD: −1.73; 95% CI: −4.07, 0.92; *p* = 0.15) (Figure 3B). Moderate heterogeneity was noted among the studies for this outcome (I^2^ = 57%), which decreased to 45% after excluding Kusunose et al. [27].

#### 3.2.6. E/A Ratio

Four studies [23,25,27,28], including 269 participants (CS: 80; ECS: 189), evaluated the E/A ratio as an outcome. No significant differences in the E/A ratio were observed between the two groups (MD: −0.11; 95% CI: −0.33, 0.12; *p* = 0.35) (Figure 3C). High heterogeneity was noted among the studies for this outcome (I^2^ = 90%). However, upon excluding Pudovattil et al., the heterogeneity decreased to 0% [28].

#### 3.2.7. E/E’ Ratio

**Figure 4:**
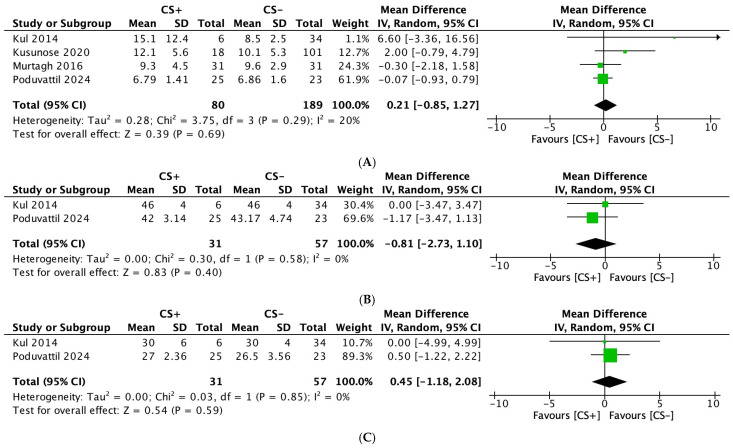
Forest plots comparing echocardiography parameters in patients with cardiac sarcoidosis (CS) to extracardiac sarcoidosis (ECS): (**A**): E/E’ ratio; (**B**): left ventricular end-diastolic diameter; and (**C**): left ventricular end-systolic diameter.

Four studies [23,25,27,28], including 269 participants (CS: 80; ECS: 189), evaluated the E/E’ ratio as an outcome. No significant differences were observed between the two groups (MD: 0.21; 95% CI: −0.85, 1.27; *p* = 0.69) (Figure 4A). Low heterogeneity was noted among the studies for this outcome (I^2^ = 20%).

#### 3.2.8. Left Ventricular End-Diastolic Diameter (mm) (LVEDD)

Two studies [23,28], including 88 participants (CS: 31; ECS: 57), evaluated the LVEDD as an outcome. No significant differences were observed between the two groups for this outcome (MD: −0.81; 95% CI: −2.73, 1.10; *p* = 0.40) (Figure 4B). No heterogeneity was noted among the studies for this outcome (I^2^ = 0%).

#### 3.2.9. Left Ventricular End-Systolic Diameter (mm) (LVESD)

Two studies [23,28], including 88 participants (CS: 31; ECS: 57), evaluated the LVESD as an outcome. No significant differences were observed between the two groups for this outcome (MD: 0.45; 95% CI: −1.18, 2.08; *p* = 0.59) (Figure 4C). No heterogeneity was noted among the studies for this outcome (I^2^ = 0%).

### 3.3. Quality Assessment of the Studies

All studies had high methodological quality as evaluated by the NOS (Supplementary Table S4). All funnel plots demonstrated minimal to low asymmetry; hence, publication bias was deemed as low for all outcomes (Supplementary Figures S1–S9).

## 4. Discussion

In this meta-analysis including patients with sarcoidosis, we explored how echocardiography parameters vary in CS vs. ECS patients. Patients who present with CS as their initial organ manifestation of sarcoidosis face a higher risk of adverse cardiac outcomes compared to those who previously had ECS. They also have shorter outcome-free survival and increased hazards of adverse cardiac events [30,31]. Therefore, enhancing awareness and improving the diagnosis of CS is crucial for earlier recognition and better management [32].

The hallmark of CS is the development of non-caseating granulomas in cardiac tissue. These granulomas are composed of diverse immune cell populations, including macrophages and multinucleated giant cells, which disrupt normal cardiac function [33,34]. Granulomas disrupt normal cardiac architecture and interfere with the heart’s electrical signaling, leading to arrhythmias such as atrioventricular block and ventricular tachycardia. This disruption is due to the remodeling of gap junctions and the absence of key proteins like plakoglobin at cell junctions [35]. The presence of granulomas and associated fibrosis can impair the heart’s ability to pump blood effectively, leading to heart failure. This is often accompanied by increased filling pressures and pulmonary pressures, indicating diastolic dysfunction [33,35]. Over time, the inflammatory process leads to fibrosis, particularly in the septum and trabeculae, further compromising cardiac function and contributing to symptoms like heart failure and arrhythmias. The focal nature of CS complicates diagnosis, as endomyocardial biopsy may yield false negatives [12,36]. Therefore, advanced imaging techniques are essential for identifying active inflammation and functional impairment for guiding treatment [37].

The cause of mortality in sarcoidosis patients is the disease itself, with pulmonary and cardiac complications being significant contributors to death [38]. CS accounts for a substantial portion of sarcoidosis-related deaths, with studies showing it to be the cause of approximately 50% of fatal cases [3]. It includes complications such as cardiac arrest, cardiomyopathy, and arrhythmias [12]. Since the presentation of CS can range from asymptomatic to causing sudden cardiac death, its early detection is crucial yet difficult [39]. The absence of specific symptoms and a gold standard diagnostic technique complicates the diagnosis. There is no single test for diagnosing CS, which leads to underdiagnosis [40]. Autopsy studies indicate a prevalence of cardiac involvement as high as 79%, contrasting with clinical registries reporting only 5% [41]. ECG and echocardiography have limited sensitivity, while advanced imaging techniques like CMR and PET are costly and not widely available [42,43]. Speckle tracking echocardiography (STE) has emerged as a valuable tool for detecting early myocardial dysfunction. STE can reveal left ventricular dysfunction not apparent in routine two-dimensional echocardiography [44]. It can detect reduced LV GLS and LV GCS in patients with normal ejection fractions, indicating early myocardial involvement. Lower strain values correlate with adverse cardiac events, aiding in risk stratification [43,45]. STE also provides a cost-effective addition to advanced imaging modalities like cardiac MRI and FDG-PET, which may not be readily available. Unlike CMR and PET, which require specialized infrastructure and longer acquisition times, STE is readily available at the bedside, making it important for routine monitoring. STE does not expose patients to ionizing radiation (as in PET) or require contrast agents (as in CMR), making it safer for patients with renal impairment or other contraindications. STE allows for the frequent and non-invasive monitoring of disease progression and treatment response, whereas repeated PET scans expose patients to cumulative radiation, and CMR may not always be feasible. STE provides detailed segmental strain analysis, offering insights into regional myocardial involvement, whereas PET focuses more on metabolic activity and CMR on structural abnormalities. STE can identify myocardial damage comparable to that detected by delayed enhancement MRI, making it a valuable tool in settings where advanced imaging is not feasible [24]. While current guidelines do not include STE for CS diagnosis, its high (47) sensitivity in detecting early myocardial dysfunction suggests that it could be a valuable addition [46]. Incorporating STE into guidelines promotes collaboration among cardiologists, electrophysiologists, and other specialists, ensuring comprehensive care for patients with CS [47].

The current landscape of CS diagnosis has various challenges. The gold standard for the diagnosis of CS is the EMB, which has inherent limitations due to the patchy nature of myocardial involvement, leading to high false-negative rates [13]. This meta-analysis highlights STE as a potential additive solution to the limitations of CMR, PET, and EMB, as it acts as a bridge between conventional echocardiography and advanced imaging modalities. STE can detect subclinical dysfunction before structural changes occur, which neither traditional echocardiography nor the LVEF can reliably detect. STE is cost-effective and widely available, making it a more practical option for repeated assessments compared to PET or CMR. Additionally, it allows for a quantitative myocardial deformation assessment, which provides an objective marker for CS-related dysfunction. This meta-analysis is notably the first systematic review consolidating STE findings in CS versus ECS, which translates to increased statistical power due to pooling data. STE could be integrated into diagnostic algorithms for CS in resource-limited settings and can also be used for longitudinal monitoring and assessing treatment response, particularly in patients undergoing immunosuppressive therapy.

GLS is a metric that measures the percentage of longitudinal shortening (the change in length relative to the baseline length). Our meta-analysis revealed a significant decrease in LV GLS in patients with CS (MD: −2.73; 95% CI: −4.09, −1.38; *p* < 0.0001) compared to patients with ECS. Studies have shown that GLS reduction often precedes a decrease in the LVEF and cannot be predicted by other echocardiographic measures [48]. LV GLS is widely used in echocardiographic strain analysis, making it a standardized and reproducible parameter in multi-center studies. A reduction in LV GLS in CS patients can have a multitude of prognostic implications. Abnormal LV GLS (<−17%) has been linked to worse clinical outcomes, including major cardiovascular events [25,46]. It can predict adverse outcomes in sarcoidosis patients, with lower values correlating with higher risks of HF and mortality [49]. Although studies have found that LV GLS is effective in detecting subclinical dysfunction, some studies say that it does not have strong prognostic value for predicting adverse outcomes specific to CS, such as ventricular arrhythmias or heart failure, as it was found to not be independently associated with these long-term outcomes in a study of patients with suspected CS [26]. These findings suggest that GLS may be a valuable tool for early screenings of subclinical CS and monitoring cardiac function in at-risk populations. LV GLS could serve as an important parameter for risk stratification, but the current guidelines do not yet incorporate LV GLS for CS. The findings of this meta-analysis support that LV GLS could be used to identify high-risk patients early before over-dysfunction develops, guide decisions on advanced therapies (e.g., implantable cardioverter-defibrillators (ICDs), aggressive immunosuppression), and monitor treatment response (improvement in LV GLS). Further research on larger cohorts is needed to clarify its role in clinical decision making for CS.

TAPSE is a crucial echocardiographic measure for assessing right ventricular (RV) systolic function, particularly in patients with sarcoidosis. While specific studies on TAPSE in sarcoidosis are limited, the existing literature highlights its significance in various cardiac conditions. TAPSE serves as a reliable indicator of RV function, correlating well with advanced imaging techniques like cardiac MRI [50]. Lower TAPSE values are associated with worse outcomes, including higher mortality and increased risk of adverse cardiac events. It is one of the most important predictors of survival, alongside other factors like the cardiac index and RV ejection fraction [51]. TAPSE provides a simple, reproducible, and load-dependent measure of RV function. Additionally, TAPSE is easily obtained using 2D echocardiography, making it widely accessible, even in resource-limited settings. CS frequently involves the RV, but standard LVEF measurements do not assess RV function. Lower RAPSE correlated with poorer outcomes, particularly in patients with pulmonary hypertension and RV failure. TAPSE is also used in conjunction with estimated pulmonary artery systolic pressure (ePASP) to assess RV-pulmonary arterial (RV-PA) coupling. In CS, RV-PA coupling tends to worsen over time, indicating progressive RV dysfunction, although ePASP may remain stable [52,53]. In patients with a reduced LVEF, TAPSE values significantly decrease, indicating RV dysfunction, as found by Tamannah et al. [54] with notable differences based on LVEF status. Given that sarcoidosis can lead to pulmonary hypertension and RV dysfunction, monitoring TAPSE may provide insights into disease progression and cardiac involvement and can guide clinical decisions, especially in assessing the severity of cardiac involvement in sarcoidosis patients [55,56,57]. Combining TAPSE with ePASP could enhance risk stratification for CS patients, such as patients with reduced TAPSE and high PASP may require closer monitoring and earlier intervention. There is an unmet need for prospective studies to establish cut-off values for such echocardiographic parameters and their integration into current CS guidelines for enhanced detection.

This meta-analysis did not find any significant difference between the two groups in terms of the LV GCS, IVST, LVEF, E/A ratio, E/E’ ratio, LVEDD, and LVESD. It could reflect the complex and multifactorial pathophysiology of sarcoidosis, where both cardiac and extracardiac manifestations may lead to subclinical myocardial alterations. Additionally, variations in study methodologies, a subset of patient populations, and echocardiographic techniques might have contributed to the lack of distinction in the LV GCS, E/A ratio, and LVEF, as the heterogeneity in this study was moderate to high.

Obesity is known to affect cardiac structure and function, including LV strain measurements, due to increased myocardial workload, subclinical fibrosis, and hemodynamic alterations [58,59]. A recent review highlights that obese individuals often have reduced GLS values, even in the absence of overt cardiac disease, which could confound the interpretation of STE findings in CS patients [60]. Given that obesity rates vary across different study populations, differences in body mass index may have influenced the observed STE differences between CS and ECS groups.

Conversely, while LVGLS is a promising tool for assessing cardiac involvement in sarcoidosis, its limitations include variability in measurement techniques and the need for further validation in larger cohorts to establish definitive cut-off values for clinical use [29,46]. STE could play a role in guiding the use of device therapies, such as ICDs, by identifying patients at high risk for SCD. This is crucial as traditional criteria may not capture all at-risk patients [61]. Additionally, STE could inform the use of immunosuppressive therapies by monitoring cardiac function and inflammation, potentially leading to more effective management strategies [62,63].

### 4.1. Future Directions

Future research should focus on standardizing STE parameters for the diagnosis and prognosis of CS. Establishing clear cut-off values for LV GLS and PALS that can reliably predict adverse outcomes or relapses will be crucial for integrating STE into routine clinical practice. Longitudinal studies are needed to assess the long-term outcomes of patients diagnosed with CS using STE. These studies should aim to correlate initial STE findings with clinical outcomes over time, providing insights into the progression of the disease and the effectiveness of different treatment strategies [46]. Most available studies on STE in CS are cross-sectional or retrospective, limiting their ability to assess disease progression and long-term outcomes. While LV GLS and TAPSE are promising prognostic markers, their ability to predict future cardiovascular events in CS has not been validated in large prospective trials. Current guidelines for CS do not yet incorporate STE for risk stratification, largely due to the lack of longitudinal outcome-based studies. Future trials should follow CS patients over multiple years to assess long-term STE prognostic implications, compare STE findings with event-driven outcome data (e.g., SCD, hospitalization, arrhythmias), and establish guideline-directed cut-off values for STE parameters in CS. Research should explore the integration of STE with other diagnostic modalities, such as FDG-PET scans, to enhance the accuracy of CS diagnosis and monitor treatment response.

To address certain ongoing challenges, recent initiatives have aimed to improve STE standardization in CS and other cardiomyopathies. The European Association of Cardiovascular Imaging (EACVI) and American Society of Echocardiography (ASE) have released guidelines emphasizing the use of vendor-neutral strain software (e.g., TomTec, EchoInsight) to improve reproducibility and adoption of standardized frame rates (50–80 fps) for accurate LV GLS measurements. AI-based strain algorithms are being developed to reduce interoperator variability and standardize GLS interpretation across imaging platforms. Future studies could integrate deep learning-based STE models for automated CS detection and risk stratification.

### 4.2. Limitations

This meta-analysis has several limitations that should be considered when interpreting the results. First, the inclusion of observational and retrospective studies contributes to moderate to high heterogeneity, which may affect the reliability and generalizability of the findings. There are various explanations for moderate to high heterogeneity in some parameters. The variability in imaging protocols and equipment (e.g., Philips iE33, GE Vivid E9/E95) leads to slight variations in strain measurements. Some studies could have also utilized vendor-specific strain algorithms, which can influence GLS values. Another explanation is that the differences in patient populations, such as CS cohorts varied in disease severity, comorbidities, and treatment status, whereas ECS controls could be heterogenous, with variations in systemic sarcoidosis burden. Subsequently, due to differences in statistical and study design (prospective vs. retrospective), the data quality and statistical power may have been affected, leading to heterogeneity in some outcomes. To evaluate the impact and to minimize the heterogeneity, we utilized standard sensitivity analyses (leave-one-out), which reinforced the reliability of the results. Second, while the sample size is sufficient, further multicenter clinical trials, as well as larger observational studies, are necessary to confirm these findings and establish more definitive conclusions. Furthermore, due to the inclusion of observational and retrospective studies, there is a risk for selection bias and confounding in this meta-analysis. Retrospective studies often include clinically diagnosed CS cases, meaning patients with subtle or subclinical CS may be underrepresented. Some studies may have excluded milder cases, skewing the results towards more severe disease presentations. Immunosuppressive therapy status was not uniformly reported—patients receiving corticosteroids or other immunomodulators may have improved cardiac function, affecting echocardiographic parameters. Differences in image acquisition and processing (frame rates, image quality, and speckle-tracking resolution) and operator-dependent variability may have contributed to inter-study differences. Third, this is a study-level meta-analysis with no reporting of individual patient-level data, which did not allow for specific confounders to be studied and adjusted. Fourth, the statistical tests for funnel plot asymmetry could not be conducted to confirm publication bias, as only seven studies were included in this meta-analysis. Fifth, the included studies did not compare STE findings against a reference standard like CMR or PET, which are currently the gold standard for diagnosing CS. Without direct validation against CMR (LGE, T2 mapping) or FDG-PET, it remains unclear whether STE findings correlate with actual myocardial inflammation or fibrosis. Future studies should incorporate direct comparisons between STE, CMR, and PET to define STE’s role within a multi-modality diagnostic framework. Sixth, the included studies did not track clinical outcomes (e.g., ventricular arrhythmias, HF progression, or SCD), limiting the ability to assess the prognostic value of STE-derived parameters. Lastly, this study only compares STE findings between CS and ECS but does not establish diagnostic accuracy, sensitivity, or specificity against gold-standard imaging. Readers should interpret the current results within the context of distinguishing cardiac vs. extracardiac involvement rather than as definitive evidence of STE’s diagnostic or prognostic power in CS.

## 5. Conclusions

While advanced imaging techniques like CMR and FDG-PET are effective, they are often limited by cost and availability. STE offers a more accessible and cost-effective alternative for early screening and ongoing management. The use of STE in conjunction with other imaging modalities like CMR and PET can facilitate a more tailored treatment approach, potentially improving patient outcomes. LV GLS and TAPSE could offer promising results in the early detection, risk stratification, and monitoring of CS. Given its ability of detect subclinical myocardial dysfunction, STE should be considered in patients with suspected CS and normal LVEF, as well as in cases where CMR or PET findings are inconclusive.

For risk stratification, LV GLS < −17% warrants closer monitoring, while LV GLS < −15% or progressive decline may indicate higher arrhythmic or HF risk, necessitating electrophysiologic evaluation and potential for ICD placement. TAPSE < 16 mm suggests RV involvement and possible pulmonary hypertension, requiring additional assessment. Despite its advantages, the standardization of STE parameters and validation of prognostic cut-offs are needed. Due to the evolving nature of this disease and its complexities, further advancements in diagnostic tools are warranted. Understanding the relationship between STE parameters and treatment response could lead to more personalized and effective management strategies for CS. The emerging technique of three-dimensional STE may further extend its clinical usefulness as a potential complementary value in CS assessment.

## Figures and Tables

**Table 1:** Baseline characteristics of the included studies.

Study	Country	Multicenter	Number of Patients (*N*)	Mean Age		Males (%)		Echocardiography System Used	History of Steroid Use		Comorbidities											
											HTN		DM		AF		CAD		Dyslipidemia		Smoking	
				CS	ECS	CS	ECS		CS	ECS	CS	ECS	CS	ECS	CS	ECS	CS	ECS	CS	ECS	CS	ECS
Kul, 2014	Turkey	No	40	45 ± 9.1	47 ± 10.3	26.4	50	Philips iE33	NR		NR		NR		0	5.8	NR		NR		NR	
Orii, 2015	Japan	No	55	64 ± 9	57 ± 13	28	27	Vivid E9 system	7	16	23	16	15%	3	NR		NR		23	16	NR	
Murtagh, 2016	United States	No	62	58.8 ± 11.3	58.4 ± 10.3	22	29	Philips iE33	71	39	52	35	29	13	13	29	16	13	32	23	13	0
Dabir, 2018	Germany	No	61	52.6 ± 10	52.7 ± 12	43	47	NR	57	63	30	16	17	8	0	3	NR		13	11	13	13
Kusunoe, 2020	Japan	No	119	68 ± 8	62 ± 13	33	32	Vivid E9/E95	NR		56	37	67	38	NR		NR		NR		NR	
Poduvattil, 2024	India	No	48	41.3 ± 12.57	46 ± 17.4	60	34.8	NR	NR		4	13	4%	8.7	NR		NR		NR		NR	
Jankowska, 2024	Poland	No	119	NR		NR		GE system	37.2	13.2	44.2	36.8	11.6	9.2	NR		NR		34.9	32.9	NR	

Reported as follows: Mean ± SD or percentage, unless indicated otherwise. Abbreviations: AF: atrial fibrillation; CAD: coronary artery disease; CS: cardiac sarcoidosis; DM: diabetes mellitus; ECS: extracardiac sarcoidosis; HTN: hypertension; and NR: not reported.

## Data Availability

No new datasets were generated during this study.

## Supplementary Materials

The following supporting information can be downloaded at: https://www.mdpi.com/article/10.3390/diagnostics15050518/s1, Supplementary Table S1. The Preferred Reporting Items for Systematic Reviews and Meta-Analyses (PRISMA) 2020 Checklist. Supplementary Table S2. Search strategy for all databases. Supplementary Table S3. Inclusion and exclusion criteria in each study. Supplementary Table S4: Quality Assessment for the included studies using the Newcastle-Ottawa Scale (NOS). Supplementary Figure S1. Funnel plot for LV GLS. Supplementary Figure S2. Funnel plot for LV GCS. Supplementary Figure S3. Funnel plot for IVST. Supplementary Figure S4. Funnel plot for TAPSE. Supplementary Figure S5. Funnel plot for LVEF. Supplementary Figure S6. Funnel plot for E/A ratio. Supplementary Figure S7. Funnel plot for E/E’ ratio. Supplementary Figure S8. Funnel plot for LVEDD. Supplementary Figure S9. Funnel plot for LVESD.

## Abbreviations

AV	Atrioventricular
CM	Cardiac magnetic resonance
CS	Cardiac sarcoidosis
E/A	Early-to-late diastolic filling velocity
E/E’	Early mitral inflow velocity to early diastolic tissue Doppler velocity
ECS	Extracardiac sarcoidosis
EMB	Endomyocardial biopsy
ePASP	Estimated pulmonary artery systolic pressure
HF	Heart failure
IVST	Interventricular septal thickness
LV GCS	Left ventricular global circumferential strain
LV GLS	Left ventricular global longitudinal strain
LVEF	Left ventricular ejection fraction
LVEDD	Left ventricular end-diastolic diameter
LVESD	Left ventricular end-systolic diameter
NOS	Newcastle–Ottawa scale
PET	Positron emission tomography
SCD	Sudden cardiac death
STE	Speckle tracking echocardiography
TAPSE	Tricuspid annular plane systolic excursion
