# Supplementary material for: Detection of Myocardial Deformation Patterns and Prognostic Value of Routine Echocardiographic Parameters in Patients with Cardiac Sarcoidosis Versus Extracardiac Sarcoidosis: Systematic Review and Meta-Analysis
Source: Diagnostics (Basel). 2025 Feb 20;15(5):518. doi: 10.3390/diagnostics15050518 (PMC11898684; doi:10.3390/diagnostics15050518)

# Supplementary Material

## Echocardiographic changes in cardiac sarcoidosis: A systematic review and meta-analysis

**Supplementary Table S1**. The Preferred Reporting Items for Systematic Reviews and Meta-Analyses (PRISMA) 2020 Checklist.

**Supplementary Table S2**. Search strategy for all databases.

**Supplementary Table S3**. Inclusion and exclusion criteria in each study.

**Supplementary Table S4:** Quality Assessment for the included studies using the Newcastle-Ottawa Scale (NOS).

**Supplementary Figure S1**. Funnel plot for LV GLS

**Supplementary Figure S2**. Funnel plot for LV GCS

**Supplementary Figure S3**. Funnel plot for IVST

**Supplementary Figure S4**. Funnel plot for TAPSE

**Supplementary Figure S5**. Funnel plot for LVEF

**Supplementary Figure S6**. Funnel plot for E/A ratio

**Supplementary Figure S7**. Funnel plot for E/E' ratio

**Supplementary Figure S8**. Funnel plot for LVEDD

**Supplementary Figure S9**. Funnel plot for LVESD

**Supplementary Table S1**. The Preferred Reporting Items for Systematic Reviews and Meta-Analyses (PRISMA) 2020 Checklist.

| Section and Topic | Item # | Checklist item | Location where item is reported |
|---|---|---|---|
| **TITLE** | | | |
| Title | 1 | Identify the report as a systematic review. | 1 |
| **ABSTRACT** | | | |
| Abstract | 2 | See the PRISMA 2020 for Abstracts checklist. | 1 |
| **INTRODUCTION** | | | |
| Rationale | 3 | Describe the rationale for the review in the context of existing knowledge. | 6 |
| Objectives | 4 | Provide an explicit statement of the objective(s) or question(s) the review addresses. | 6, 7 |
| **METHODS** | | | |
| Eligibility criteria | 5 | Specify the inclusion and exclusion criteria for the review and how studies were grouped for the syntheses. | 8, 9 |
| Information sources | 6 | Specify all databases, registers, websites, organisations, reference lists and other sources searched or consulted to identify studies. Specify the date when each source was last searched or consulted. | 8, 9 |
| Search strategy | 7 | Present the full search strategies for all databases, registers, and websites, including any filters and limits used. | 8 |
| Selection process | 8 | Specify the methods used to decide whether a study met the inclusion criteria of the review, including how many reviewers screened each record and each report retrieved, whether they worked independently, and if applicable, details of automation tools used in the process. | 8, 9 |
| Data collection process | 9 | Specify the methods used to collect data from reports, including how many reviewers collected data from each report, whether they worked independently, any processes for obtaining or confirming data from study investigators, and if applicable, details of automation tools used in the process. | 8, 9 |
| Data items | 10a | List and define all outcomes for which data were sought. Specify whether all results that were compatible with each outcome domain in each study were sought (e.g. for all measures, time points, analyses), and if not, the methods used to decide which results to collect. | 8, 9 |
| | 10b | List and define all other variables for which data were sought (e.g. participant and intervention characteristics, funding sources). Describe any assumptions made about any missing or unclear information. | 8, 9 |
| Study risk of bias assessment | 11 | Specify the methods used to assess risk of bias in the included studies, including details of the tool(s) used, how many reviewers assessed each study and whether they worked independently, and if applicable, details of automation tools used in the process. | 9, 10 |

| | | | |
|---|---|---|---|
| Effect measures | 12 | Specify for each outcome the effect measure(s) (e.g. risk ratio, mean difference) used in the synthesis or presentation of results. | 9, 10 |
| Synthesis methods | 13a | Describe the processes used to decide which studies were eligible for each synthesis (e.g. tabulating the study intervention characteristics and comparing against the planned groups for each synthesis (item #5)). | 10 |
| | 13b | Describe any methods required to prepare the data for presentation or synthesis, such as handling of missing summary statistics, or data conversions. | 10 |
| | 13c | Describe any methods used to tabulate or visually display results of individual studies and syntheses. | 10 |
| | 13d | Describe any methods used to synthesize results and provide a rationale for the choice(s). If meta-analysis was performed, describe the model(s), method(s) to identify the presence and extent of statistical heterogeneity, and software package(s) used. | 10 |
| | 13e | Describe any methods used to explore possible causes of heterogeneity among study results (e.g. subgroup analysis, meta-regression). | 10 |
| | 13f | Describe any sensitivity analyses conducted to assess robustness of the synthesized results. | 10 |
| Reporting bias assessment | 14 | Describe any methods used to assess risk of bias due to missing results in a synthesis (arising from reporting biases). | NR |
| Certainty assessment | 15 | Describe any methods used to assess certainty (or confidence) in the body of evidence for an outcome. | NR |
| **RESULTS** | | | |
| Study selection | 16a | Describe the results of the search and selection process, from the number of records identified in the search to the number of studies included in the review, ideally using a flow diagram. | 11 |
| | 16b | Cite studies that might appear to meet the inclusion criteria, but which were excluded, and explain why they were excluded. | 11 |
| Study characteristics | 17 | Cite each included study and present its characteristics. | 11 |
| Risk of bias in studies | 18 | Present assessments of risk of bias for each included study. | 14 |
| Results of individual studies | 19 | For all outcomes, present, for each study: (a) summary statistics for each group (where appropriate) and (b) an effect estimate and its precision (e.g. confidence/credible interval), ideally using structured tables or plots. | 11, 12, 13, 14 |
| Results of syntheses | 20a | For each synthesis, briefly summarise the characteristics and risk of bias among contributing studies. | 11, 12, 13, 14 |
| | 20b | Present results of all statistical syntheses conducted. If meta-analysis was done, present for each the summary estimate and its precision (e.g. confidence/credible interval) and measures of statistical heterogeneity. If comparing groups, describe the direction of the effect. | 11, 12, 13, 14 |
| | 20c | Present results of all investigations of possible causes of heterogeneity among study results. | 11, 12, 13, 14 |

| | | | |
|---|---|---|---|
| | 20d | Present results of all sensitivity analyses conducted to assess the robustness of the synthesized results. | 11, 12, 13, 14 |
| Reporting biases | 21 | Present assessments of risk of bias due to missing results (arising from reporting biases) for each synthesis assessed. | NR |
| Certainty of evidence | 22 | Present assessments of certainty (or confidence) in the body of evidence for each outcome assessed. | NR |
| **DISCUSSION** | | | |
| Discussion | 23a | Provide a general interpretation of the results in the context of other evidence. | 15, 16, 17, 18, 19 |
| | 23b | Discuss any limitations of the evidence included in the review. | 19 |
| | 23c | Discuss any limitations of the review processes used. | 19 |
| | 23d | Discuss implications of the results for practice, policy, and future research. | 18, 19 |
| **OTHER INFORMATION** | | | |
| Registration and protocol | 24a | Provide registration information for the review, including register name and registration number, or state that the review was not registered. | 8 |
| | 24b | Indicate where the review protocol can be accessed, or state that a protocol was not prepared. | 8 |
| | 24c | Describe and explain any amendments to information provided at registration or in the protocol. | 8 |
| Support | 25 | Describe sources of financial or non-financial support for the review, and the role of the funders or sponsors in the review. | Title page |
| Competing interests | 26 | Declare any competing interests of review authors. | Title page |
| Availability of data, code and other materials | 27 | Report which of the following are publicly available and where they can be found: template data collection forms; data extracted from included studies; data used for all analyses; analytic code; any other materials used in the review. | Title page |

**Supplementary Table S2**. Search strategy for all databases.

| **No.** | **Database** | **Search Strategy** | **Number of Articles** |
|---|---|---|---|
| 1. | PubMed | (cardiac sarcoidosis) AND ((echocardiography) OR (left ventricular ejection fraction) OR (doppler echocardiography) OR (speckle tracking) OR (strain imaging)) | 747 |
| 2. | Embase | (cardiac sarcoidosis) AND ((echocardiography) OR (left ventricular ejection fraction) OR (doppler echocardiography) OR (speckle tracking) OR (strain imaging)) | 619 |
| 3. | Web of Science | (TS= (“cardiac sarcoidosis”)) AND (TS= (“echocardiography”) OR TS= (“left ventricular ejection fraction”) OR TS= (“doppler echocardiography”) OR TS= (“speckle tracking”) OR TS= (“strain imaging”)) | 477 |
| 4. | Scopus | ((TITLE-ABS-KEY (cardiac sarcoidosis)) AND (TITLE-ABS-KEY (echocardiography) OR TITLE-ABS-KEY (left ventricular ejection fraction) OR TITLE-ABS-KEY (doppler echocardiography) OR TITLE-ABS-KEY (speckle tracking) OR TITLE-ABS-KEY (strain imaging))) | 542 |
| 5. | Cochrane | (MeSH descriptor: [Cardiac Sarcoidosis] explode all trees) AND ((MeSH descriptor: [echocardiography] explode all trees) OR (“Sarcoidosis”)) AND ((MeSH descriptor: [Myocardial deformation] explode all trees) OR (MeSH descriptor: [left ventricular strain] explode all trees)) | 73 |
| TOTAL | | | 2458 |

**Supplementary Table S3:** Inclusion and exclusion criteria in each study.

| Study | Inclusion criteria | Exclusion Criteria |
|---|---|---|
| Kul 2014 | - Known history of:<br>- Hypertension (HT).<br>- Diabetes mellitus (DM).<br>- Heart failure (HF).<br>- Atrial fibrillation (AF).<br>- coronary artery disease (CAD).<br>- More than mild valvular disorders.<br>- Or the presence of an implanted pacemaker or defibrillator.<br>- Thyroid disease<br>- Liver disease<br>- renal disease. | - Biopsy-proven sarcoidosis (PS) of grade 1 or 2.<br>- Diagnosis confirmed histopathologically using ATS/ERS consensus criteria.<br>- Potential extra-pulmonary and extracardiac involvement (e.g., skin, eyes, lymph nodes).<br>- Healthy subjects matched by age, body mass index (BMI), heart rate, and blood pressure. |
| Orii 2015 | - Biopsy-proven extracardiac sarcoidosis.<br>- Healthy control subjects:<br>- Age-matched.<br>- No evidence of sarcoidosis, coronary artery disease, or valvular disease.<br>- No noncardiac comorbidities such as hepatic, renal, or malignant diseases. | - Clinical findings of cardiac involvement, including:<br>- Left ventricular dysfunction (LVEF < 50%).<br>- Abnormal electrocardiographic findings (atrioventricular block, ventricular arrhythmias, complete bundle branch block, axis deviation, abnormal Q wave).<br>- Abnormal echocardiographic findings (wall thinning, regional abnormal wall motion, ventricular aneurysm).<br>- Presence of atrial fibrillation.<br>- Contraindications to MRI (severe renal impairment, implantable cardioverter-defibrillator, or pacemaker). |
| Murtagh 2016 | - Biopsy-proven extracardiac sarcoidosis referred for CMR and TTE to evaluate for cardiac sarcoidosis (CS).<br>- Preserved left ventricular ejection fraction (LVEF > 50%).<br>- Patients who were in sinus rhythm during imaging (even if they had a history of CAD or AF)<br>- Matched controls (CS-group) based on age, sex, and severity of pulmonary sarcoidosis using Scadding stage and pulmonary function test (PFT) parameters.<br>- At least 1 year data was present. | - LVEF < 50%.<br>- CMR and TTE performed more than 12 months apart.<br>- Inadequate TTE image quality. |
| Dabir 2018 | - Biopsy-proven sarcoidosis.<br>- Underwent cardiac magnetic resonance imaging (CMR) for inflammatory cardiac disease assessment.<br>- Cardiac sarcoidosis diagnosed based on CMR findings:<br>- Increased left ventricular early gadolinium enhancement ratio (EGEr ≥4).<br>- Increased myocardial signal on T2- | - History of significant cardiac disease (e.g., myocardial infarction, myocarditis, cardiomyopathy) except arterial hypertension. |

| | | |
|---|---|---|
| | weighted images (T2-ratio ≥2).<br>- Presence of at least one myocardial lesion with non-ischemic late gadolinium enhancement (LGE).<br>- An age-matched group of healthy volunteers served as controls. | |
| Kusunoe 2020 | - Patients with confirmed sarcoidosis who underwent echocardiographic evaluation for cardiac sarcoidosis (CS).<br>- Diagnosis of CS defined using histological or clinical diagnosis criteria per Japanese Ministry of Health and Welfare (JMHW) guidelines.<br>- Sarcoidosis patients without cardiac involvement.<br>- Advanced imaging tests (radionuclide scanning, cardiac magnetic resonance imaging, or both) performed at baseline.<br>- Age- and gender-matched control patients recruited from a volunteer database with comprehensive histories and physical examinations.<br>- All examinations (laboratory, echocardiographic, and advanced cardiovascular imaging) completed within one month. | - Pre-existing structural heart diseases.<br>- Poor echocardiographic images. |
| Poduvattil 2024 | - Preexisting cardiac disorders (coronary artery disease, heart failure, cardiomyopathy, known left ventricular dysfunction).<br>- Chronic kidney disease (CKD).<br>- Chronic liver disease (CLD).<br>- Already receiving immunosuppressive therapy. | - Newly diagnosed sarcoidosis patients aged >15 years.<br>- Histopathological diagnosis of noncaseating granulomas, ruling out all other potential diagnoses. |
| Jankowska 2024 | - Patients with sarcoidosis screened for cardiac involvement using the HRS expert consensus approach.<br>- Screening methods included clinical symptoms, ECG, echocardiography, 24-hour Holter monitoring, speckle tracking echocardiography, and cardiac magnetic resonance imaging (CMR).<br>- Histopathological confirmation of sarcoidosis.<br>- Speckle tracking echocardiography and CMR performed within 0–6 months. | - Incomplete data.<br>- Sarcoidosis unconfirmed in histopathological examination.<br>- Conditions potentially interfering with strain or late gadolinium enhancement (LGE) assessment:<br>- coronary artery disease (CAD).<br>- Left bundle branch block.<br>- Valvular heart disease.<br>- Hypertrophic cardiomyopathy.<br>- Atrioventricular block and permanent right ventricular pacing. |

**Supplementary Table S4:** Quality Assessment for the included studies using the Newcastle-Ottawa Scale (NOS).

| **Study ID** | **Selection** | | | | **Comparability** | **Outcome** | | | **Total Score** |
|---|---|---|---|---|---|---|---|---|---|
| | Representativeness of the exposed cohort | Selection of the non-exposed cohort | Ascertainment of exposure | Outcome of interest was not present at the start of the study | | Assessment of outcome | Follow-up duration | Adequacy of follow-up | |
| Kul 2014 | ☆ | ☆ | ☆ | ☆ | ☆ | ☆ | ☆ | ☆ | 8 |
| Orii 2015 | ☆ | ☆ | ☆ | ☆ | ☆ | ☆ | ☆ | ☆ | 8 |
| Murtagh 2016 | ☆ | ☆ | ☆ | ☆ | ☆☆ | ☆ | ☆ | ☆ | 9 |
| Dabir 2018 | ☆ | | ☆ | ☆ | ☆☆ | ☆ | ☆ | ☆ | 7 |
| Kusunose 2020 | ☆ | ☆ | ☆ | ☆ | ☆☆ | ☆ | ☆ | ☆ | 9 |
| Poduvattil 2024 | ☆ | ☆ | ☆ | ☆ | ☆ | ☆ | ☆ | ☆ | 8 |
| Jankowska 2024 | ☆ | ☆ | ☆ | ☆ | ☆ | ☆ | ☆ | ☆ | 8 |

**Supplementary Figure S1**. Funnel plot for LV GLS

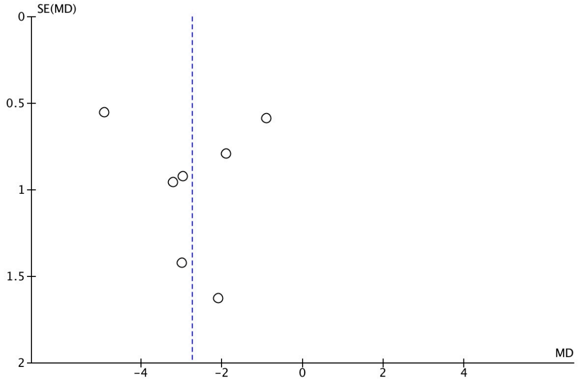

**Supplementary Figure S2**. Funnel plot for LV GCS

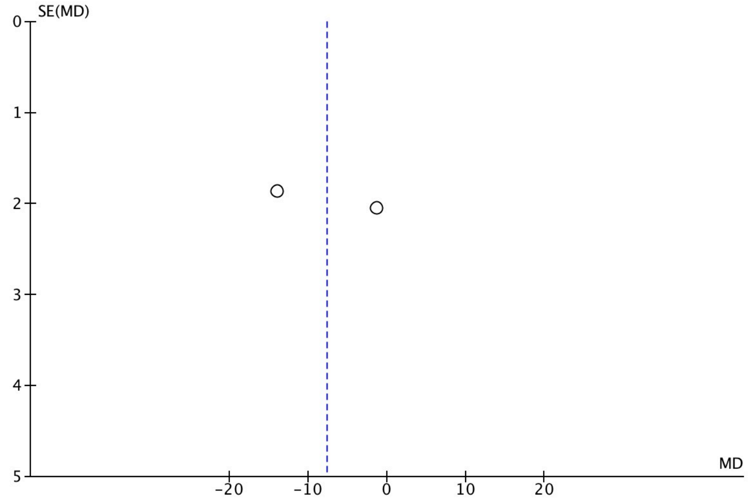

**Supplementary Figure S3**. Funnel plot for IVST

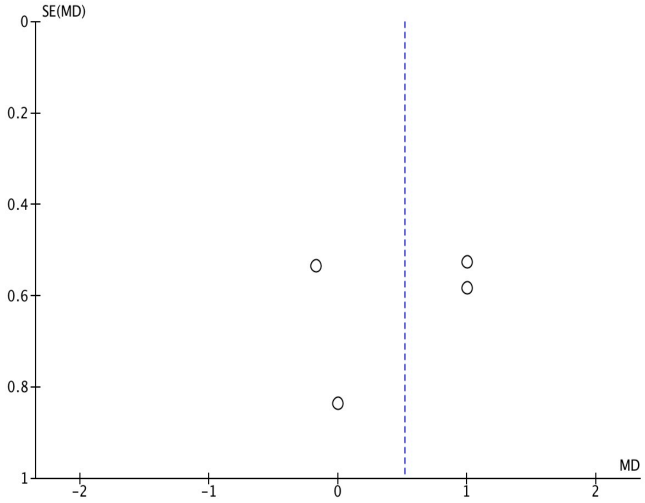

**Supplementary Figure S4**. Funnel plot for TAPSE

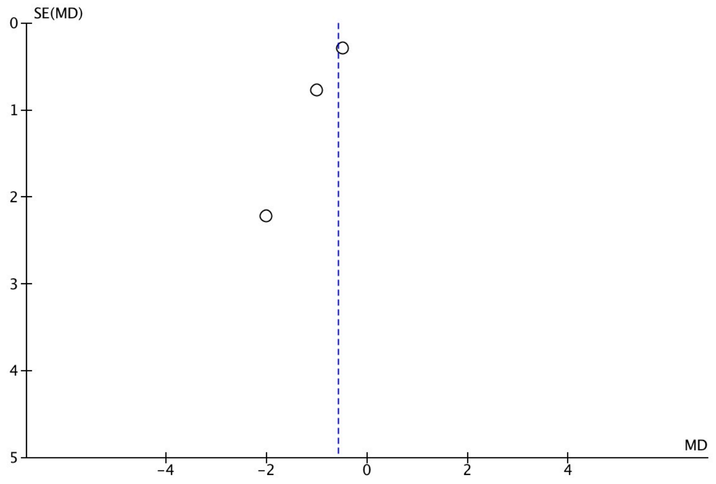

**Supplementary Figure S5**. Funnel plot for LVEF

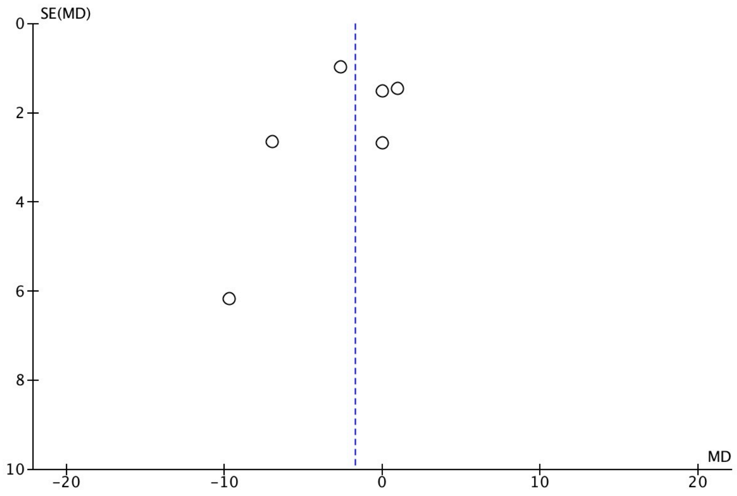

**Supplementary Figure S6**. Funnel plot for E/A ratio

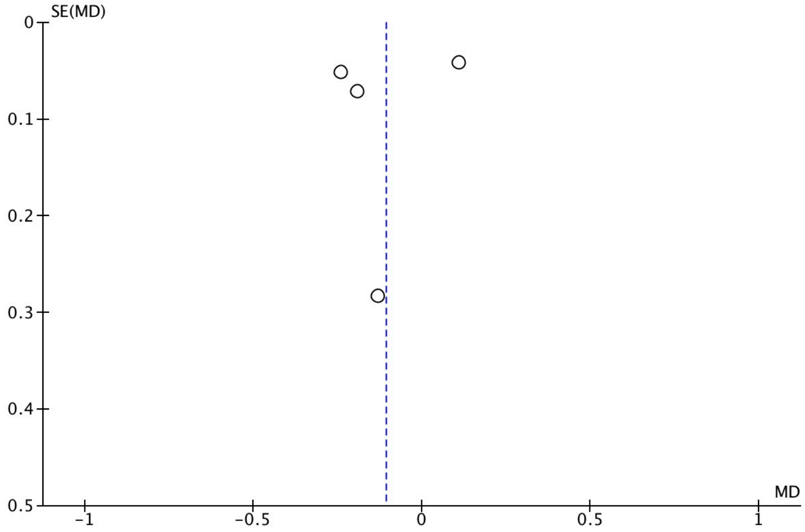

**Supplementary Figure S7**. Funnel plot for E/E’ ratio

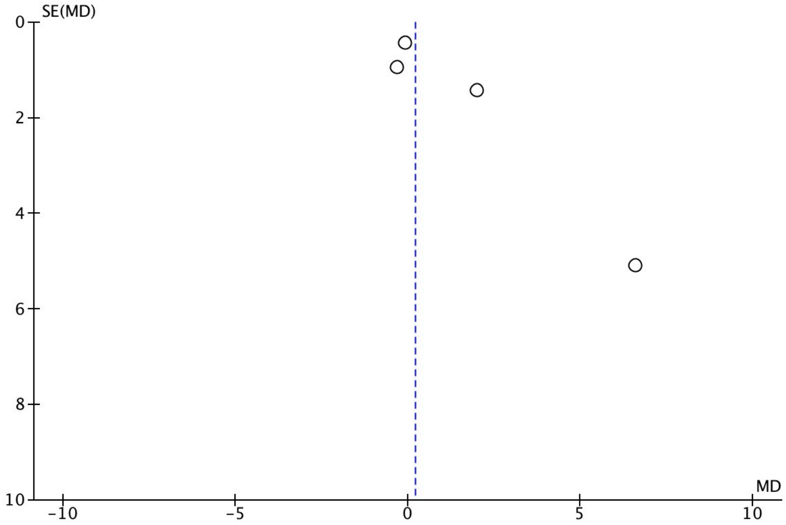

**Supplementary Figure S8**. Funnel plot for LVEDD

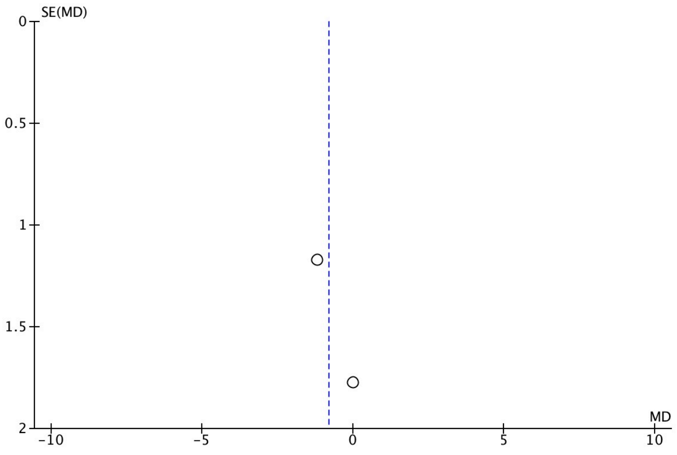

**Supplementary Figure S9**. Funnel plot for LVESD

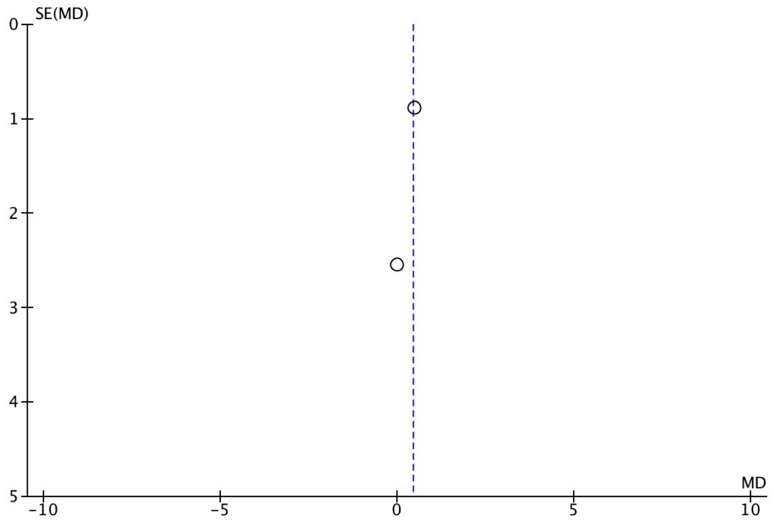

Supplement: Supplementary file 1 [file diagnostics-15-00518-s001.zip › diagnostics-3461537-supplementary.pdf]
